# Gastric peroral endoscopic myotomy through an altered anatomy with presternal oesocoloplasty

**DOI:** 10.1055/a-2974-0900

**Published:** 2026-10-15

**Authors:** Maxime Amoyel, Gabriel Marcellier, Paul Rivallin, Bénédicte Jais, Alain Berson, David Giannetta, Frédéric Prat

**Affiliations:** 1 Gastroenterology 55076 Hôpital Bichat - Claude-BernardParisFrance; 2 UFR de Médecine 72790 Université de Paris UFR de Médecine de Paris CentreParisÎle-de-FranceFrance; 3 Endoscopy Unit 55100 Beaujon HospitalClichyÎle-de-FranceFrance

We report the case of a 65-year-old man with a history of pre-sternal ileocoloplasty following carcinologic oesophagogastrectomy with preservation of the antrum ( Fig. 1 ). The patient had developed dysphagia and severe bloating of his presternal plasty ( Fig. 2 ). Self-administered massage of the subcutaneous swelling was no longer sufficient to empty the plasty.

**Fig. 1:**
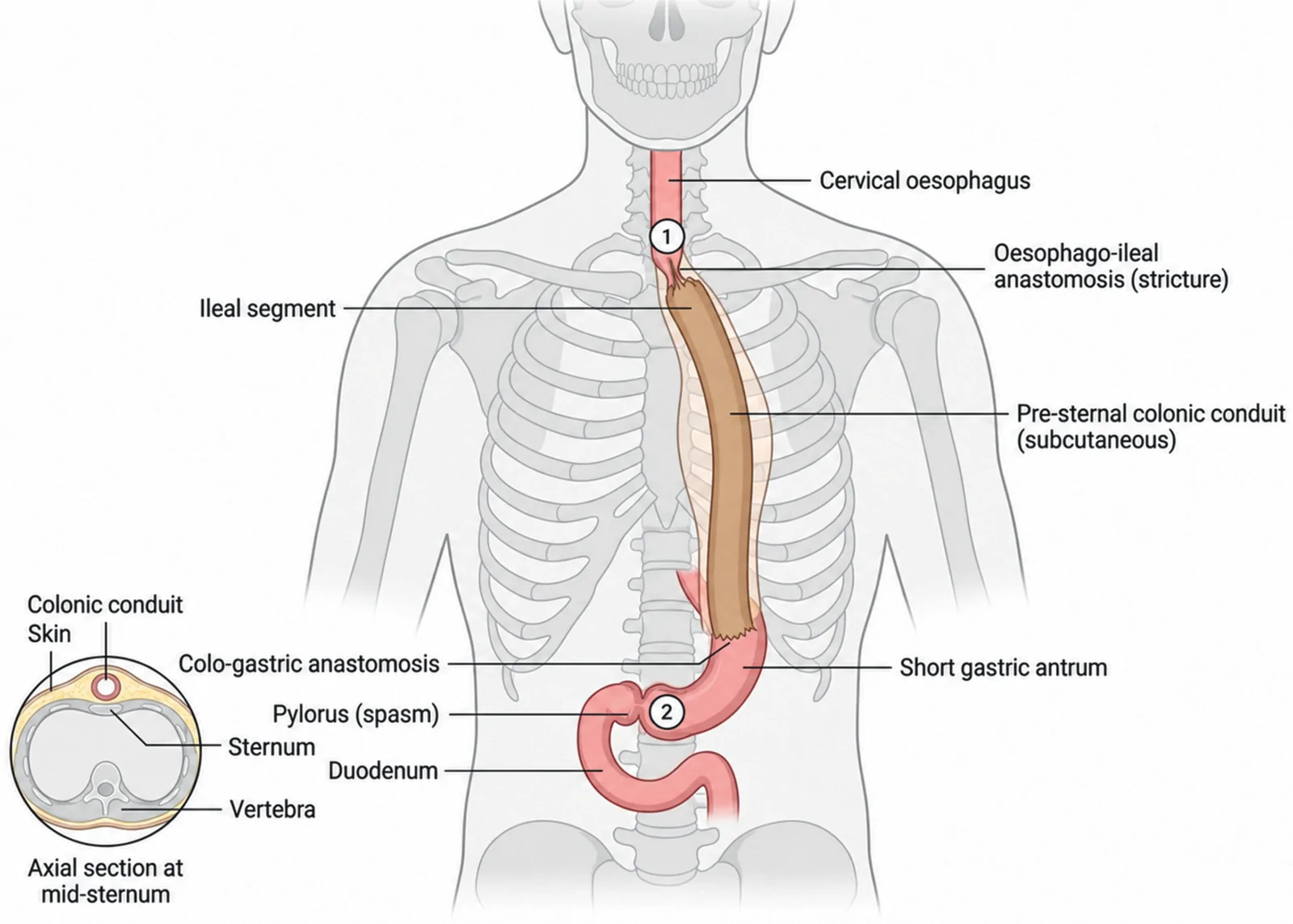
Schematic representation of the reconstructed foregut anatomy. After carcinologic oesophagogastrectomy with antral preservation, continuity was restored by an ileocolic graft tunneled subcutaneously in front of the sternum. The proximal ileal segment is anastomosed to the cervical oesophagus, and the distal colonic segment is anastomosed to the retained short gastric antrum. Two levels of obstruction were identified: (1) a tight oesophago-ileal anastomotic stricture, treated by hydrostatic balloon dilation to 18 mm; (2) a refractory pylorospasm, treated by gastric per-oral endoscopic myotomy. An axial section at the mid-sternum level shows the conduit lying subcutaneously in front of the sternum. Source: [rerif] ﻿created in BioRender. Amoyel, M. (2026) https://BioRender.com/ haw9023 1​

**Fig. 2:**
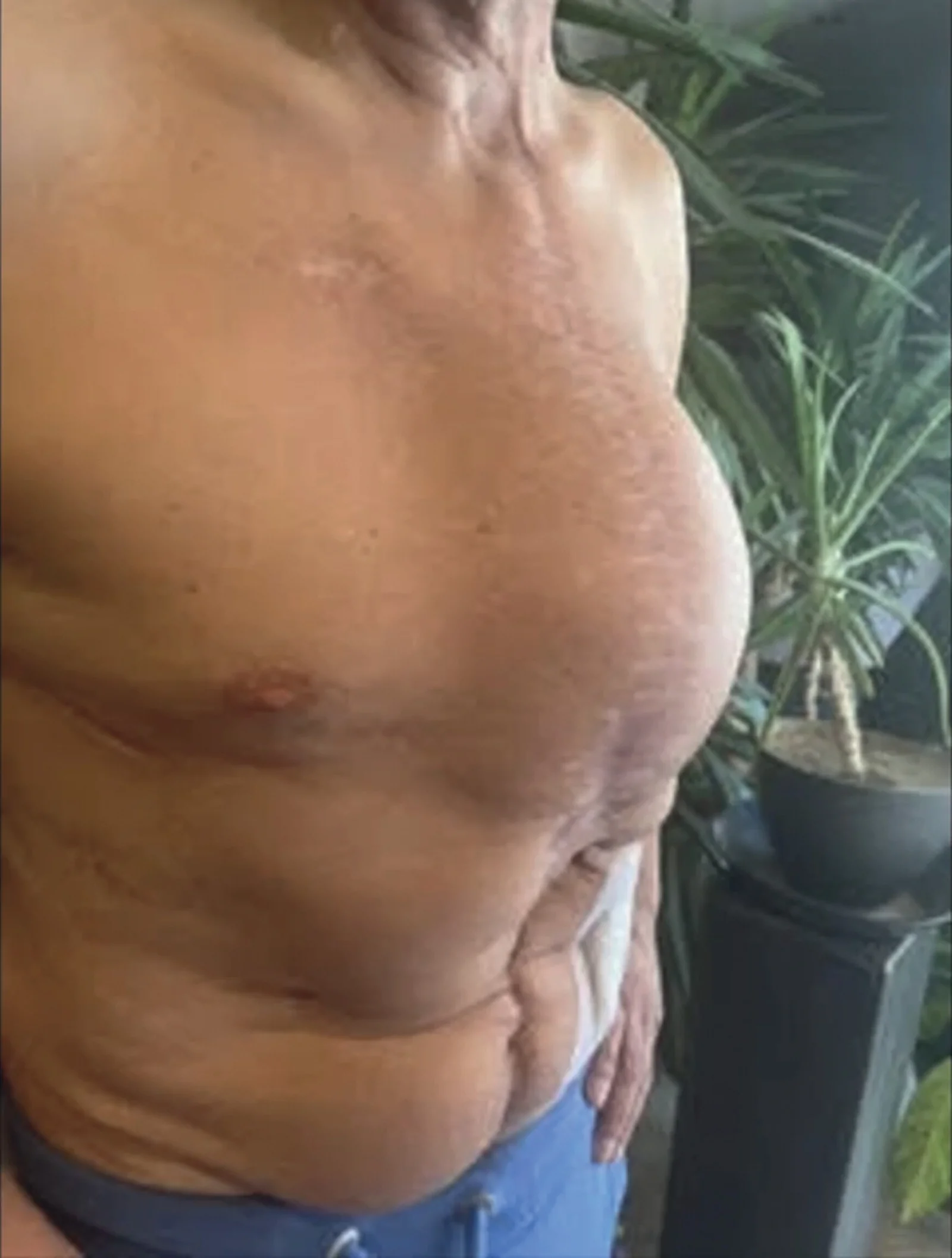
Clinical appearance of the pre-sternal ileocolic conduit after a meal, showing marked subcutaneous bloating of the plasty in front of the sternum. This visible swelling provided a simple clinical marker of impaired emptying and was used to monitor symptomatic improvement.

A tight oesoileal upper anastomotic stricture had been previously treated several times by hydrostatic dilation. A refractory pylorospasm, related to the gastric denervation with a very short antrum, was deemed responsible for the post-prandial swelling of the pre-sternal coloplasty and provided a clinical marker to monitor symptomatic improvement and to disentangle the respective contributions of the stricture and the pylorospasm.

Endoscopy confirmed the two levels of obstruction: at the upper anastomosis, a tight stricture ( Fig. 3 ); at the colo-gastric anastomosis, a very short antrum with a pylorospasm impassable to the endoscope.

**Fig. 3:**
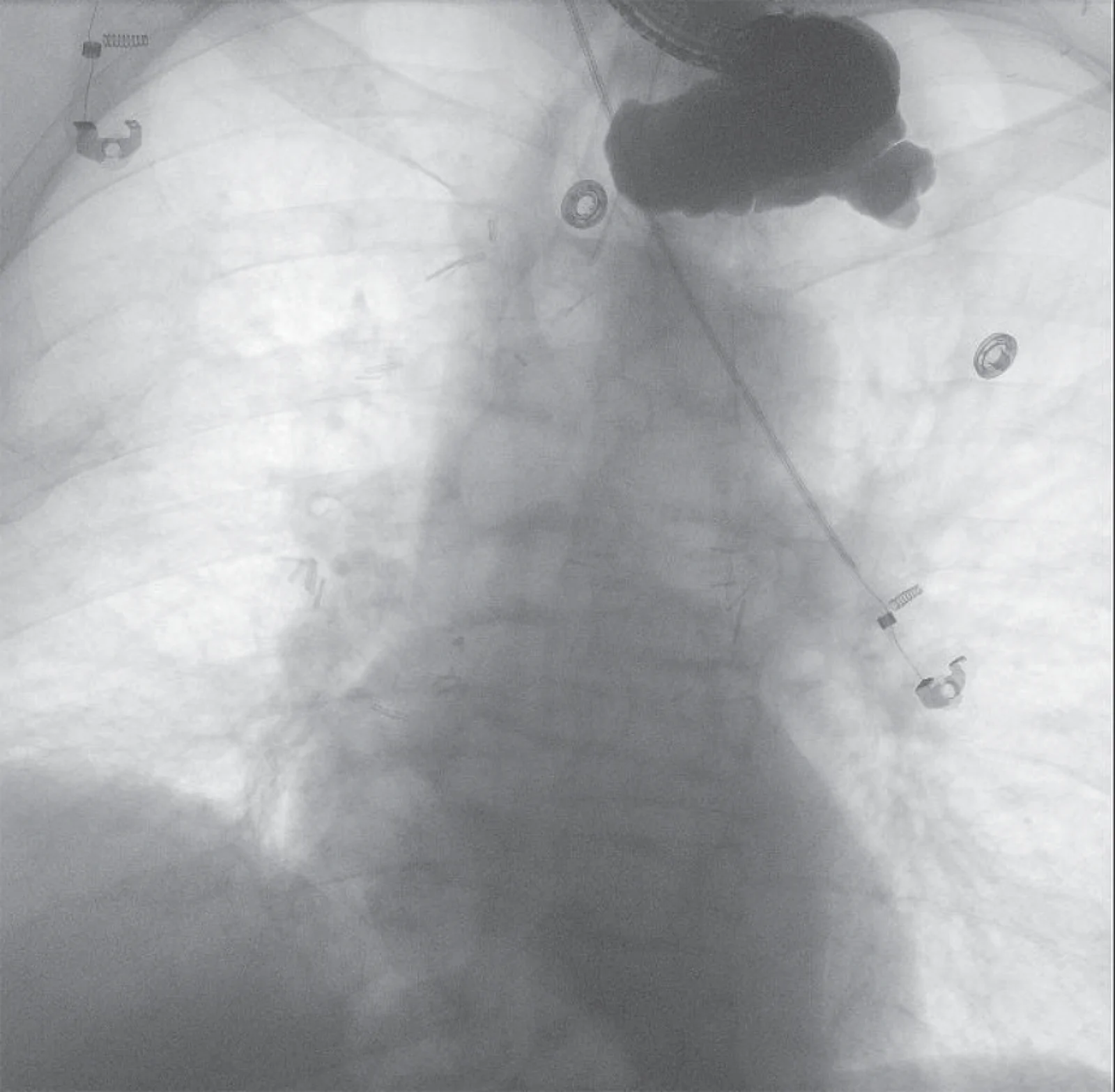
A fluoroscopic view of the pre-sternal ileocolic conduit after contrast injection, showing the tight oesophagoileal anastomotic stricture with retention of contrast above the narrowing.

We first performed a hydrostatic balloon dilation of the oesoileal anastomotic stricture up to 18 mm ( Fig. 4 ). In the same session, a gastric peroral endoscopic myotomy (G-POEM) was carried out: submucosal injection, mucosal incision, submucosal tunneling to the pyloric region, full pyloric myotomy and antral myotomy over 25 mm, and closure of the mucosal entry with clips. At the end of the procedure, the endoscope passed freely through the pylorus, confirming loosening of the pylorospasm ( Video 1 ). No intra- or post-procedural adverse events occurred. No post-procedural contrast study was performed. Both presenting symptoms resolved: oral intake became unrestricted, and the pre-sternal plasty no longer became bloated after meals.

**Fig. 4:**
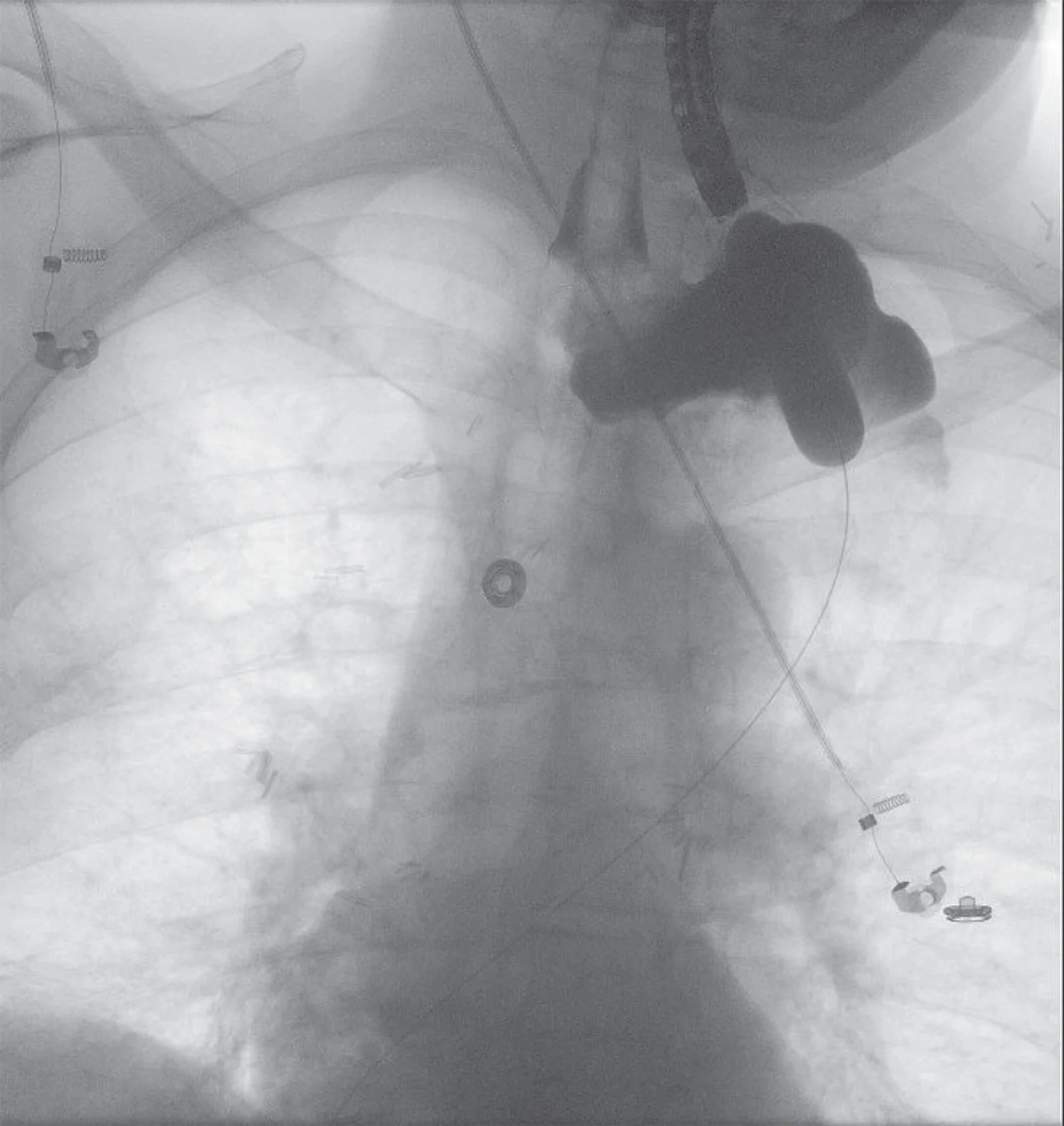
A fluoroscopic view of the hydrostatic balloon dilation of the oesophagoileal anastomosis to 18 mm.

**Video 1** Hydrostatic dilation and G-POEM in a 65-year-old man with an oesoileal anastomotic stricture and refractory pylorospasm after oesophagectomy with pre-sternal ileocoloplasty and preservation of the distal stomach. Video link below: .


G-POEM with or without hydrostatic dilation appears to be a safe and effective rescue option to restore luminal patency and relieve refractory pylorospasm, even in patients with severely altered foregut anatomy such as a presternal ileocoloplasty.

Endoscopy_UCTN_Code_TTT_1AO_2AG_3AZ

## Declaration of GenAI Use

Generative artificial intelligence (AI; Claude, Anthropic) was used to assist with English language editing and phrasing of the manuscript. No generative AI was used for the analysis or interpretation of the clinical data, for the creation or modification of any image or video, or for the drafting of scientific content.
